# Quality and reliability of femoral neck fracture educational short videos: a cross-sectional study

**DOI:** 10.1038/s41598-026-46431-y

**Published:** 2026-03-30

**Authors:** Jiakuan Tu, Shuihua Xie, Jiaxin Zheng, Zhe Lin, Wang Ren

**Affiliations:** 1 Department of Orthopedics, Jiangxi Province Hospital of Integrated Chinese and Western Medicine, Nanchang, Jiangxi China; 2 Jiangxi University of Chinese Medicine, Nanchang, 330004 Jiangxi China

**Keywords:** DISCERN tool, Femoral neck fractures, Global quality score, Health information quality, Short-video platforms, FNF-SCCS, PEMAT-A/V, Health care, Medical research

## Abstract

Background: Femoral neck fracture (FNF) patients increasingly use short-video platforms (TikTok, Bilibili) for education, but content quality and reliability are underexplored. Objective: To systematically evaluate FNF-related educational short videos on TikTok and Bilibili, and explore associations between video characteristics, user engagement, and quality scores. Methods: This cross-sectional study analyzed 166 top-ranked videos (accessed in May 2025 as guest users) using modified DISCERN, Global Quality Score (GQS), the newly developed Femoral Neck Fracture-Specific Clinical Comprehensiveness Score (FNF-SCCS), and Patient Education Materials Assessment Tool for Audiovisual Materials (PEMAT-A/V). Videos were categorized by uploader type and content theme; non-parametric statistics, Cohen’s kappa, and Intraclass Correlation Coefficient (ICC) assessed associations and inter-rater reliability. Results: Professionals created 59.04% of content, non-professionals 25.9%. Bilibili videos were longer (162s vs. 59s, *P* < 0.05) and higher-quality (GQS ≥ 4: 43.7% vs. 12.6%; DISCERN ≥ 4: 40.9% vs. 23.2%, *P* < 0.05). Disease knowledge and rehabilitation training videos scored highest (DISCERN = 4, GQS = 4), personal experiences lowest (DISCERN = 2, GQS = 2). FNF-SCCS (ICC = 0.91) and PEMAT-A/V (ICC = 0.89) showed excellent inter-rater reliability; professional institutions/individuals outperformed non-professionals in FNF-SCCS and PEMAT understandability (Median = 75.0% vs. 58.3%, *P* < 0.001), while overall actionability was low except for rehabilitation training videos (Median = 100.0%). Engagement weakly correlated with quality (*r*=-0.21–0.08); Cohen’s kappa = 0.806–0.839. Conclusions: FNF-related content on TikTok and Bilibili exhibits suboptimal quality, with professional sources outperforming non-professional ones. Critical gaps exist in guideline adherence and actionable instructions. Platforms and healthcare professionals should optimize medical content dissemination.

**Supplementary Information:**

The online version contains supplementary material available at 10.1038/s41598-026-46431-y.

## Introduction

Femoral neck fractures (FNF), a prevalent and debilitating condition among the elderly, are associated with high morbidity and mortality rates, often termed the “last fracture” due to their severe impact on patient outcomes^1–3^. As the global population ages, the incidence of FNF continues to rise^4^, increasing the demand for accessible and trustworthy health information. Short-video platforms have emerged as one of the primary sources of patient education for FNF, but this role necessitates the provision of accessible and accurate educational resources to address the growing needs of aging populations^5^.

TikTok and Bilibili—two of China’s most widely used platforms—host millions of health-related videos that offer easily digestible content to a broad audience^6^.Notably, approximately half of the adult population consults the internet for health-related information, a behavior that underscores the pivotal role of short-video platforms in health communication, especially for populations seeking health guidance before medical care^7^. What’s more, well-structured and trustworthy information(the causes, pathophysiology, treatment and prevention of a disease, etc.) can help patients learn about their diseases, understand treatment options, and implement preventive measures^8^. For FNF—a condition requiring strict adherence to rehabilitation protocols and long-term management strategies—inadequate or misleading information from low-quality videos may hinder patient compliance, delay recovery, or even increase the risk of complications, while high-quality educational content can empower patients to make informed decisions and collaborate effectively with healthcare providers.

Furthermore, videos integrate not only verbal narration but also substantial written content, which is closely linked to health literacy. Recognizing this, authoritative bodies including the National Institutes of Health^9^, the US Department of Health and Human Services (HHS), and the American Medical Association (AMA) have established readability standards for patient education materials, recommending a Grade 6 level to ensure accessibility and comprehension by the general public^10^. Despite the significant role of these platforms and the clear readability requirements, the quality and reliability of FNF-related content remain questionable: studies reveal widespread inaccuracies in health-related videos, a lack of scientific rigor, and a predominance of non-professional creators, raising concerns about misinformation^11^. For orthopedic topics, which require precise dissemination of surgical techniques, rehabilitation protocols, and long-term management strategies, these gaps could have direct implications for patient care. For example, some studies have found that non-professional videos frequently contain unverified treatment claims or oversimplified explanations of complex procedures^12,13^, highlighting the need for systematic quality assessments.

Against this backdrop, this study aims to evaluate FNF educational content on TikTok and Bilibili. Distinct from previous research relying solely on generic metrics (DISCERN/GQS), we address the current limitations by introducing a disease-specific clinical rubric (FNF-SCCS) based on AAOS/NICE guidelines and the validated PEMAT-A/V tool. This approach allows for a pioneering analysis of clinical guideline adherence, audiovisual understandability, and actionability. Our findings pinpoint critical blind spots in digital health communication, offering actionable insights for healthcare providers, platform regulators, and patients to improve health literacy and clinical outcomes for FNF.

## Methods

### Search and data collection

**Fig. 1:**
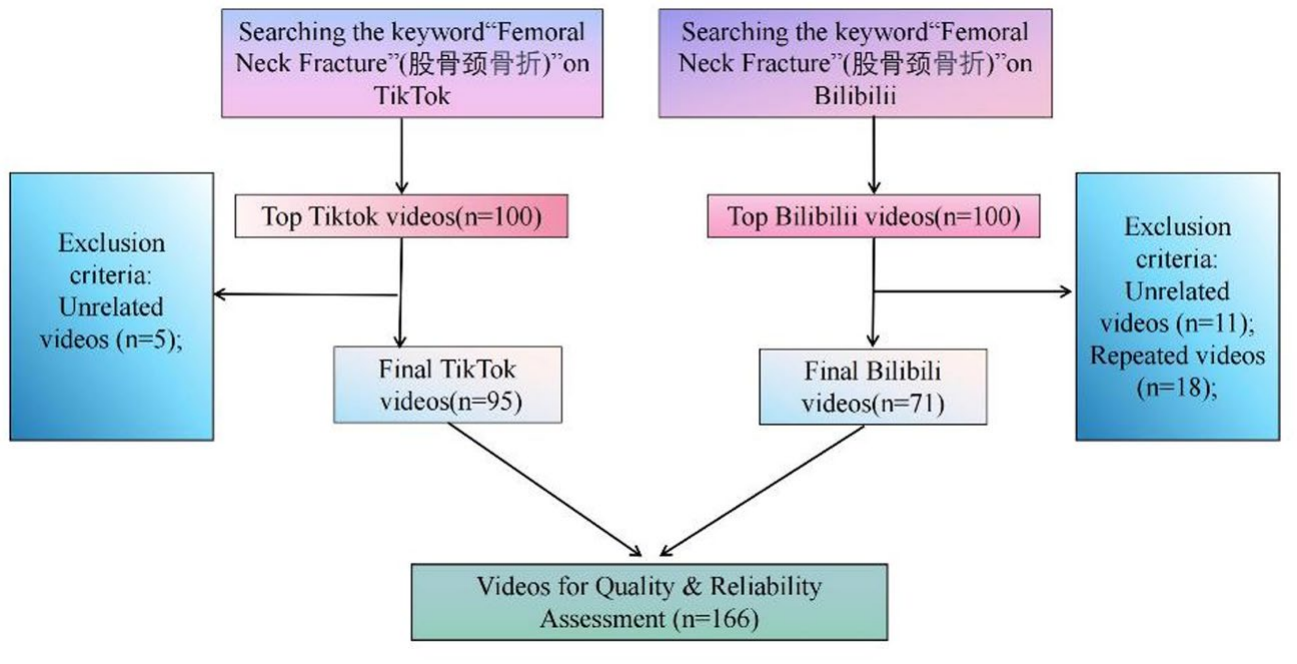
Search strategy and video filtering protocol.

In the present study, “Femoral Neck Fracture” was adopted as the search term. A total of 100 top-ranked videos were retrieved from TikTok, with an equivalent number obtained from Bilibili, all videos were accessed in May 2025. This ensures that content published before May 2025 is included. The measures taken to minimize bias: all videos were accessed as “guest users” (unlogged-in accounts) to avoid personalized recommendations. Subsequently, duplicate videos and those irrelevant to the research objectives were strictly excluded (5 videos removed from the TikTok dataset and 29 from the Bilibili dataset, as shown in Fig. 1). Our analysis focused on the top 100 videos, as prior studies confirm that videos outside this range have minimal analytical impact^14^. Details of the selected videos—including title, uploader’s occupation, content, duration, and engagement metrics (likes, collections, shares)—were documented, and all extracted data were systematically recorded in a Microsoft Excel spreadsheet.

### Video categorization

Videos were categorized into four groups by uploader type:

1. Professional individuals: Those with formal medical/healthcare qualifications (licensed physicians, physical therapists, and so on) or relevant academic backgrounds (e.g., orthopedic medical researchers)
2. Non-professional individuals: Those without formal medical/healthcare training (e.g., FNF patients sharing experiences, non-credentialed fitness enthusiasts)
3. Professional institutions: Organizations with explicit medical/healthcare mandates (e.g., hospitals, academic medical centers, national health agencies).
4. Non-professional institutions: Organizations not primarily focused on healthcare (e.g., general media, fitness brands, non-medical community groups).

Based on content, videos were further classified into:

1. Disease knowledge (e.g., FNF etiology, symptoms, diagnostic criteria);
2. Rehabilitation training (non-surgical femoral neck fracture interventions: exercise therapy, physical modalities, bone-healing lifestyle adjustments).
3. Personal experience.
4. Treatment (e.g., pharmacological therapies, surgical options).

### Assessment methodologies

In this study, the reliability of the information was evaluated using the modified DISCERN tool, while the overall quality of the videos was assessed using the Global Quality Score (GQS).

Modified DISCERN tool (Supplementary File 2) was used to assess the overall reliability of video content. The DISCERN instrument, widely recognized in academic research, is frequently employed to help consumers and healthcare providers evaluate the quality of health-related information^15,16^. It evaluates video content across five specific criteria: clarity (clarity, conciseness, and comprehensibility), relevance (pertinence to the topic), traceability (citation of valid sources), robustness (comprehensiveness and balance of information), and fairness (unbiased presentation). Each criterion is scored dichotomously (“yes” = 1 point, “no” = 0 points), yielding total scores ranging from 0 to 5 to quantify reliability.

We employed the Global Quality Score (GQS; Supplementary File 1) ^17^ to assess the overall quality of video content. Widely recognized as a tool for evaluating the reliability and quality of health-related information on digital video platforms, the GQS uses a 5-point scale: a score of 1 indicates low quality, while a score of 5 signifies superior quality.

To address the limitations of generic tools (DISCERN and GQS) in evaluating subject-specific clinical depth and guideline adherence, we developed the Femoral Neck Fracture-Specific Clinical Comprehensiveness Score (FNF-SCCS) (Supplementary File 3). Based on core clinical guidelines from the American Academy of Orthopaedic Surgeons (AAOS) and the National Institute for Health and Care Excellence (NICE)^18,19^, this 5-item rubric evaluates crucial domains: (1) early surgery timing, (2) early postoperative mobilization, (3) venous thromboembolism (VTE) prophylaxis, (4) secondary prevention for osteoporosis, and (5) the absence of medical misinformation. Each domain is scored on a 3-point scale (0 = unmentioned/incorrect, 1 = mentioned but incomplete, 2 = accurate and comprehensive), yielding a total score ranging from 0 to 10.

Furthermore, to rigorously assess the “readability” and practical utility of the audiovisual content, we utilized the Patient Education Materials Assessment Tool for Audiovisual Materials (PEMAT-A/V) (Supplementary File 4)^20^, a validated gold-standard instrument developed by the Agency for Healthcare Research and Quality (AHRQ). Videos were evaluated across 17 items to calculate two distinct percentage scores: Understandability (the extent to which viewers can process and explain key messages) and Actionability (the extent to which viewers are provided with explicit instructions to take action).

### Statistical analysis

Due to the non-parametric data distribution, values are reported as median and interquartile range (IQR). Pairwise group comparisons use the Mann–Whitney test, and the Kruskal–Wallis H test is applied for three or more groups (such as comparing FNF-SCCS and PEMAT scores across different uploader types). Fisher’s exact test compares GQS and DISCERN scores. Inter-rater agreement is evaluated via Cohen’s kappa coefficient, while the Intraclass Correlation Coefficient (ICC) was utilized to assess inter-rater reliability for continuous and ordinal scoring systems (FNF-SCCS and PEMAT-A/V). Given the non-normal data distribution, Spearman correlation analysis will assess associations among video-related variables and their relationships with scores. Statistical significance is defined as *P* < 0.05.

### Assessment protocol

The video evaluations were conducted by two senior orthopedic physicians (reviewer A and reviewer B) from the Department of Orthopedics at a top-tier tertiary teaching hospital in China. Their assessments were performed according to standardized guidelines for Femoral Neck Fracture (FNF) and strictly followed the predefined codebooks for all four assessment tools (GQS, DISCERN, FNF-SCCS, and PEMAT-A/V). In cases of disagreement between the two evaluators, consensus was achieved through discussions with an orthopedic expert with over 40 years of clinical experience. Inter-rater consistency was quantitatively analyzed to validate the reliability of the results.

## Result

### Video characteristics and uploader types

**Fig. 2:**
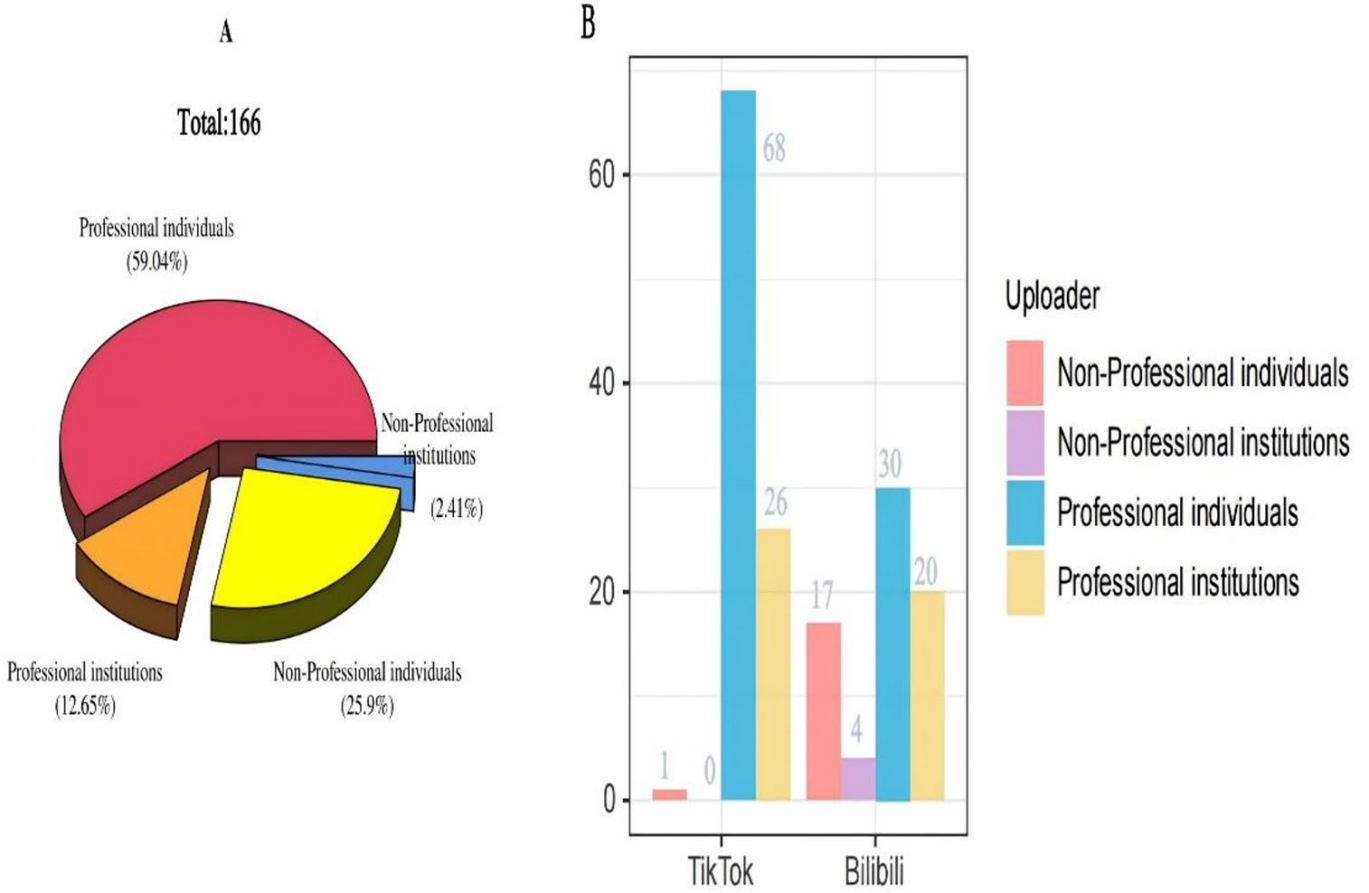
FNF content uploader analysis: (**A**) Platform-wide uploader distribution (3D pie); (**B**) Platform-specific uploader percentages (bar plot).

We analyzed 166 videos from Bilibili and TikTok, of which 71 (42.8%) were in the Bilibili group and 95 (57.2%) in the TikTok group. TikTok videos demonstrated significantly higher median values for likes (327.0 vs. 22.0), collections (69.0 vs. 23.0), and shares (45.0 vs. 11.0) compared to Bilibili (all *P* < 0.05). Conversely, the median video duration was longer in Bilibili (162.0 s) than in TikTok (59.0 s), with statistical significance (*P* < 0.05). Table 1 summarizes the baseline characteristics of the videos. Among the 166 videos, professional individuals were the predominant uploaders, constituting 59.04% of the total. Non-professional individuals accounted for 25.9%, professional institutions 12.65%, and non-professional institutions 2.41% in Fig. 2). This finding indicates that professionals are predominantly responsible for creating and disseminating videos related to FNF. Furthermore, TikTok featured a skewed distribution, with professional individuals uploading 68 videos (71.6% of TikTok’s total), followed by professional institutions (27.4%, *n* = 26). Non-professional entities contributed negligibly (0.95%, *n* = 1). Bilibili exhibited more balanced distribution: professional individuals (42.3%, *n* = 30), professional institutions (28.2%, *n* = 20), non-professional individuals (23.9%, *n* = 17), and non-professional institutions (5.6%, *n* = 4) in Fig. 2. Moreover, in Table 2, median likes varied significantly across groups (overall: 130.5; non-professional individuals: 159.0; non-professional institutions: 36.0; professional individuals: 176.0; professional institutions: 18.0; with statistical significance *P* < 0.001). No between-group differences were observed for collections. Shared counts differed among groups (overall: 29.5; non-professional individuals: 32.0; non-professional institutions: 106.5; professional individuals: 35.5; professional institutions: 10.0; *P* < 0.05). Duration also differed significantly (overall: 90.5; non-professional individuals: 98.0; non-professional institutions: 206.0; professional individuals: 64.5; professional institutions: 341.0; *P* < 0.001).

**Table 1:** Content Attributes of TikTok and Bilibili Videos.

Variables	Total (*n* = 166)	Bilibili (*n* = 71)	TikTok (*n* = 95)	*P*
Likes, M (Q₁, Q₃)	130.50 (33.00, 463.00)	22.00 (6.50, 76.00)	327.00 (135.00, 838.50)	**< 0.001**
Collections, M (Q₁, Q₃)	47.00 (13.50, 166.00)	23.00 (6.00, 73.50)	69.00 (21.00, 256.50)	**< 0.001**
Shared, M (Q₁, Q₃)	29.50 (9.00, 128.25)	11.00 (6.00, 53.50)	45.00 (16.50, 263.00)	**< 0.001**
Duration(S), M (Q₁, Q₃)	90.50 (48.00, 193.00)	162.00 (83.00, 357.00)	59.00 (39.50, 104.50)	**< 0.001**

**Table 2:** Classification of Video Uploaders on TikTok and Bilibili.

Variables	Total (*n* = 166)	Non-Professional individuals (*n* = 43)	Non-Professional institutions (*n* = 4)	Professional individuals (*n* = 98)	Professional institutions (*n* = 21)	*P*
Likes, M (Q₁, Q₃)	130.50 (33.00, 463.00)	159.00 (66.00,506.50)	36.00 (35.00,47.25)	176.00 (34.00,551.50)	18.00 (6.00,75.00)	**< 0.001**
Collections, M (Q₁, Q₃)	47.00 (13.50, 166.00)	39.00 (20.00,203.00)	62.50 (54.75,82.75)	51.00 (12.00,215.25)	21.00 (8.00,69.00)	0.262
Shared, M (Q₁, Q₃)	29.50 (9.00, 128.25)	32.00 (10.00,137.50)	106.50 (76.00,138.25)	35.50 (10.00,148.25)	10.00 (7.00,22.00)	**0.031**
Duration(S), M (Q₁, Q₃)	90.50 (48.00, 193.00)	98.00 (57.50,154.00)	206.00 (164.50,274.00)	64.50 (41.00,109.00)	341.00 (211.00,657.00)	**< 0.001**

**Fig. 3:**
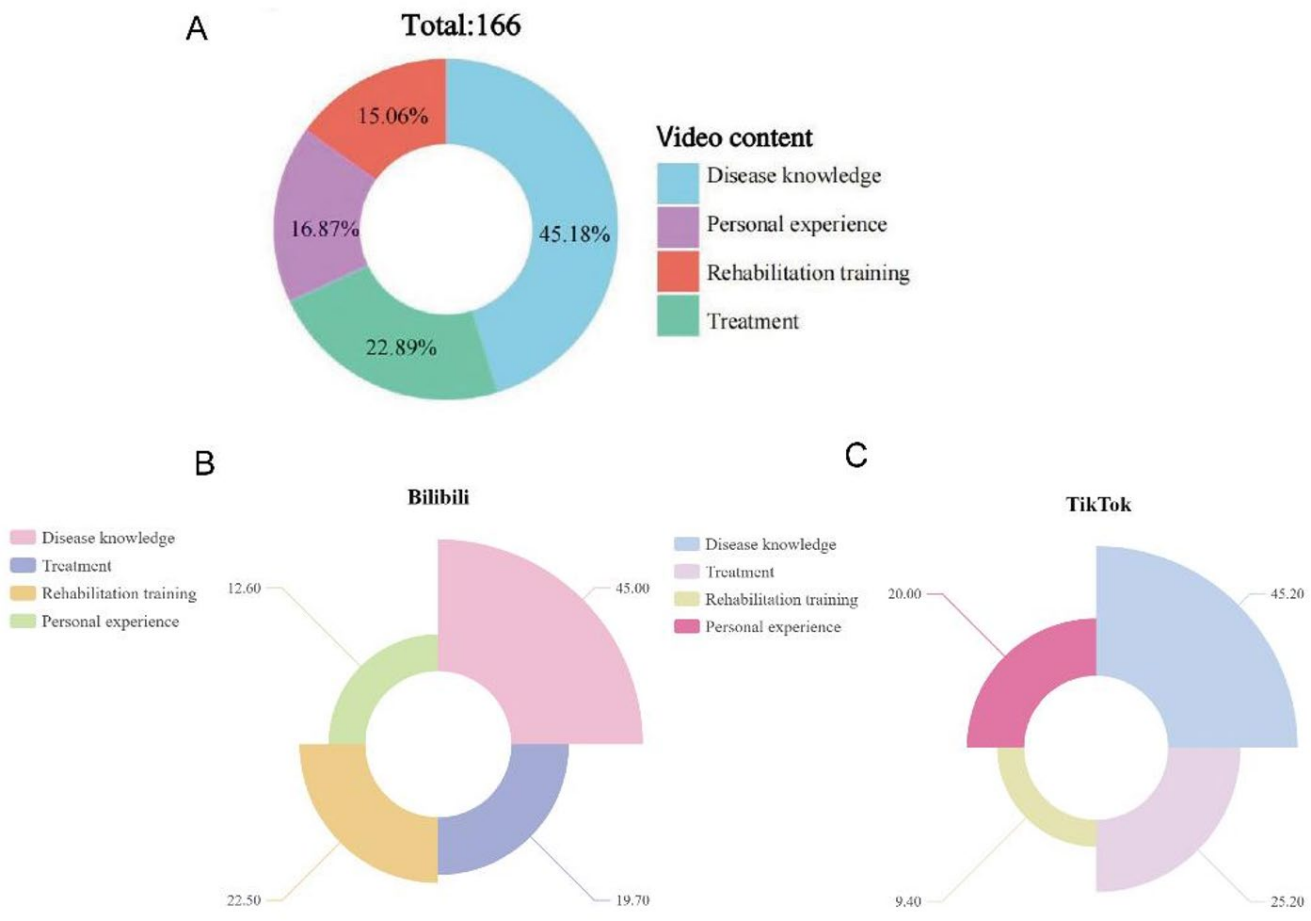
FNF video content analysis: (**A**) Cross-platform content distribution (donut chart ); (**B**) TikTok content breakdown (Basic Nightingale Rose Chart in percentage); (**C**) Bilibili content breakdown (Basic Nightingale Rose Chart in percentage).

To further explore content-specific differences in video characteristics and user engagement, we analyzed variables across four content themes in Table 3. For engagement metrics, personal experience videos had the highest median likes (176.50), followed by treatment-related videos (149.50), while rehabilitation training videos had the lowest median likes (84.00); however, these differences were not statistically significant (*P* = 0.376). Similarly, no significant between-group variations were observed in collections (*P* = 0.476) or shares (*P* = 0.300), though rehabilitation training videos tended to have higher median shares (84.00) than other themes.

**Table 3:** Content attributes of TikTok and bilibili videos.

Variables	Total (*n* = 166)	Disease knowledge (*n* = 75)	personal experience (*n* = 28)	Rehabilitation training (*n* = 25)	Treatment (*n* = 38)	*P*
Likes, M (Q₁, Q₃)	130.50 (33.00, 463.00)	130.00 (25.00,368.50)	176.50 (71.50,789.50)	84.00 (33.00,311.00)	149.50 (26.25,540.50)	0.376
Collections, M (Q₁, Q₃)	47.00 (13.50, 166.00)	41.00 (14.00,114.50)	37.50 (20.00,189.50)	69.00 (31.00,233.00)	45.00 (8.00,176.50)	0.476
Shared, M (Q₁, Q₃)	29.50 (9.00, 128.25)	20.00 (9.50,93.00)	29.00 (9.00,81.50)	84.00 (13.00,215.00)	31.00 (6.25,113.00)	0.300
Duration(M), M (Q₁, Q₃)	0.04 (0.03, 0.07)	0.05 (0.03,0.07)	0.05 (0.03,0.07)	0.09 (0.04,0.14)	0.03 (0.03,0.04)	0.062

### GQS and DISCERN score distribution across platforms

**Fig. 4:**
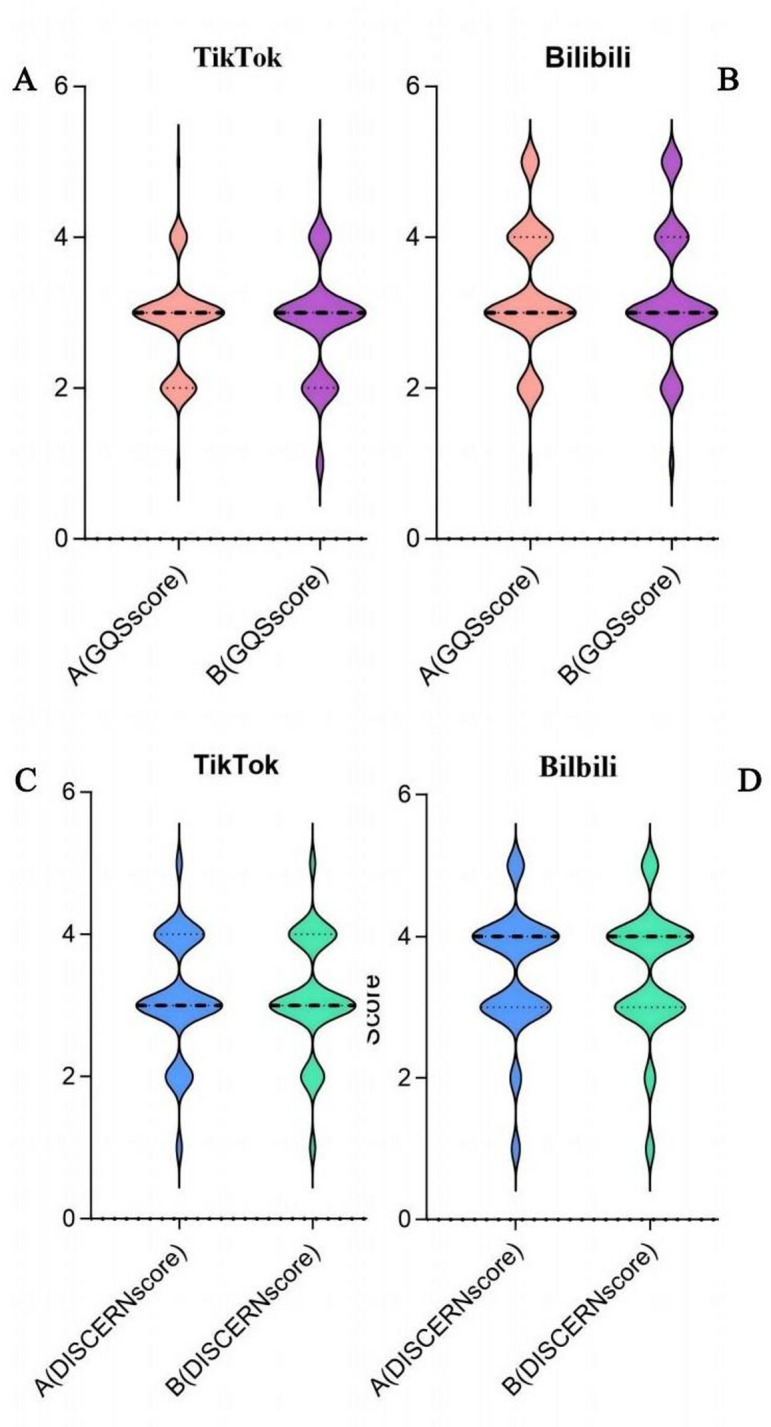
GQS and DISCERN scores for FNF videos on both platforms(Violin plot ): (**A**) TikTok GQS score distribution; (**B**) Bilibili GQS score distribution; (**C**) TikTok DISCERN score distribution; (**D**) Bilibili DISCERN score distribution.

Figure 4 shows violin plots of GQS and DISCERN scores from two reviewers comparing TikTok and Bilibili.For GQS in TikTok: Reviewer A clustered at the median; Reviewer B showed wider dispersion (partial overlap). Bilibili has a similar overlap; Reviewer A is central, Reviewer B is more dispersed (positive skew). Also, in DISCERN Scores, TikTok has shown that Reviewer A median-centered; Reviewer B is more variable (wider range, prominent tails). Bilibili suggests that Reviewer A has a tight median clustering (2–4 range); Reviewer B is more dispersed.

Table 4 compares scoring patterns between platforms. For the GQS scores, Reviewer A observed significant differences (*p* < 0.001): high scores (4–5) were more prevalent on Bilibili (43.7%) than TikTok (12.6%), while mid-range scores^21^ dominated TikTok (62.1% vs. 42.3% on Bilibili). Similarly, Reviewer B’s GQS assessment (*p* < 0.001) showed Bilibili had a higher proportion of top scores (5: 15.5% vs. 1.1% on TikTok). For DISCERN scores, Reviewer A noted disparities (*p* = 0.017), with Bilibili participants more frequently awarding 4 (40.9% vs. 23.2%) and TikTok participants favoring 3 (54.7% vs. 40.9%). Reviewer B’s evaluation (*p* = 0.018) mirrored this trend, with Bilibili again exhibiting higher top scores (4–5: 53.5% vs. 29.5%).

**Table 4:** GQS and DISCERN Ratings of TikTok and Bilibili Videos by Reviewers.

Reviewers’ score	Total (*n* = 166)	Bilibili (*n* = 71)	TikTok (*n* = 95)	*P*
A(GQSscore), n(%)				**< 0.001**
1	2 (1.20)	1 (1.41)	1 (1.05)	
2	32 (19.28)	9 (12.68)	23 (24.21)	
3	89 (53.61)	30 (42.25)	59 (62.11)	
4	33 (19.88)	22 (30.99)	11 (11.58)	
5	10 (6.02)	9 (12.68)	1 (1.05)	
B(GQSscore), n(%)				**< 0.001**
1	6 (3.61)	2 (2.82)	4 (4.21)	
2	29 (17.47)	7 (9.86)	22 (23.16)	
3	89 (53.61)	34 (47.89)	55 (57.89)	
4	30 (18.07)	17 (23.94)	13 (13.68)	
5	12 (7.23)	11 (15.49)	1 (1.05)	
A(DISCERNscore), n(%)				**0.017**
1	6 (3.61)	3 (4.23)	3 (3.16)	
2	19 (11.45)	4 (5.63)	15 (15.79)	
3	81 (48.80)	29 (40.85)	52 (54.74)	
4	51 (30.72)	29 (40.85)	22 (23.16)	
5	9 (5.42)	6 (8.45)	3 (3.16)	
B(DISCERNscore), n(%)				**0.018**
1	6 (3.61)	3 (4.23)	3 (3.16)	
2	17 (10.24)	4 (5.63)	13 (13.68)	
3	77 (46.39)	26 (36.62)	51 (53.68)	
4	57 (34.34)	32 (45.07)	25 (26.32)	
5	9 (5.42)	6 (8.45)	3 (3.16)	

Table 5 presents score variations among uploader types. Reviewer A’s median GQS score was 3.00 overall, with significant group differences (*P* < 0.001): non-professional individuals (2.00), non-professional institutions (3.50), professional individuals (3.00), and professional institutions (4.00). Reviewer B’s GQS scores showed similar variation (median = 3.00; *P* < 0.001). For DISCERN scores, both reviewers demonstrated significant group differences (*P* < 0.001): Reviewer A (median = 3.00) and Reviewer B (median = 3.00, IQR: 3.00–4.00).

**Table 5:** Evaluations by various commenters for diverse uploader types on TikTok and Bilibili.

Reviewers’ score	Total (*n* = 166)	Non-Professional individuals (*n* = 43)	Non-Professional institutions (*n* = 4)	Professional individuals (*n* = 98)	Professional institutions (*n* = 21)	*P*
A(GQSscore), M (Q₁, Q₃)	3.00 (3.00, 4.00)	2.00 (2.00,3.00)	3.50 (3.00,4.00)	3.00 (3.00,3.00)	4.00 (4.00,5.00)	**< 0.001**
B(GQSscore), M (Q₁, Q₃)	3.00 (3.00, 3.75)	2.00 (2.00,3.00)	3.00 (3.00,3.25)	3.00 (3.00,3.00)	4.00 (4.00,5.00)	**< 0.001**
A(DISCERNscore), M (Q₁, Q₃)	3.00 (3.00, 4.00)	2.00 (2.00,3.00)	3.00 (3.00,3.25)	3.00 (3.00,4.00)	4.00 (3.00,4.00)	**< 0.001**
B(DISCERNscore), M (Q₁, Q₃)	3.00 (3.00, 4.00)	3.00 (2.00,3.00)	3.00 (3.00,3.25)	3.00 (3.00,4.00)	4.00 (3.00,4.00)	**< 0.001**

**Fig. 5:**
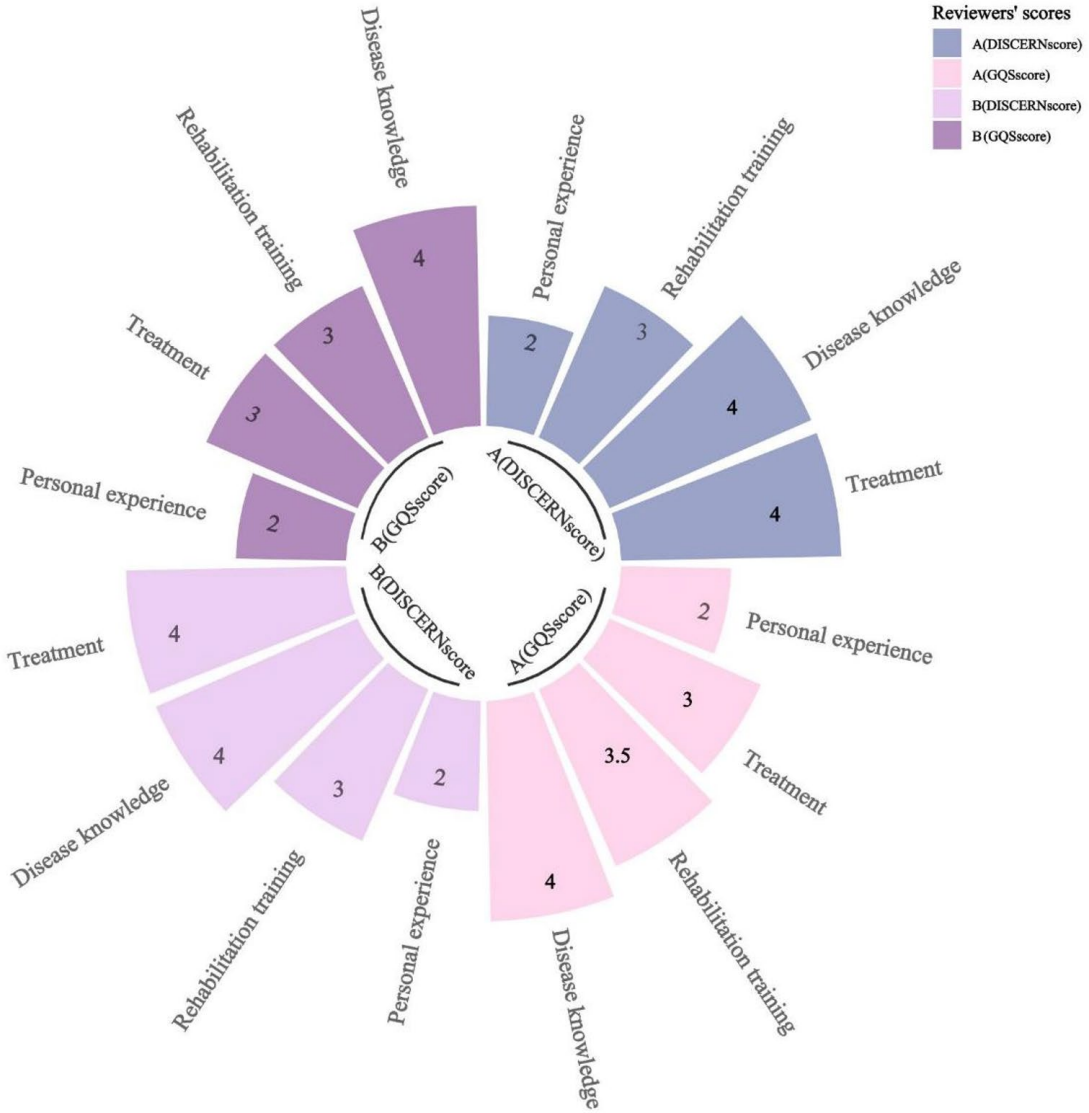
Circular Barplot showing the average scores obtained for the different video contents.

In addition, Fig. 5 analyzes the average scores obtained for the different video contents. Both reviewers gave disease knowledge maximum scores (DISCERN = 4, GQS = 4). Treatment protocols showed scoring discrepancies: Reviewer A: DISCERN, GQS = 3, while Reviewer B: DISCERN = 3, GQS = 3. Rehabilitation training achieved uniformly high scores DISCERN = 4, GQS = 4), indicating robust quality standards. Personal experiences scored lowest (DISCERN = 2, GQS = 2), reflecting significant quality limitations in scientific rigor.

### Reliability of data

To assess the interrater reliability comprehensively, Cohen’s kappa coefficient was utilized. For the Global Quality Score (GQS) and DISCERN tool, the calculated kappa coefficients were 0.839 and 0.806, respectively. These results demonstrated a high level of agreement between raters for all evaluated items, indicating substantial consistency in their assessments across the relevant criteria.

### Spearman correlation analysis

**Fig. 6:**
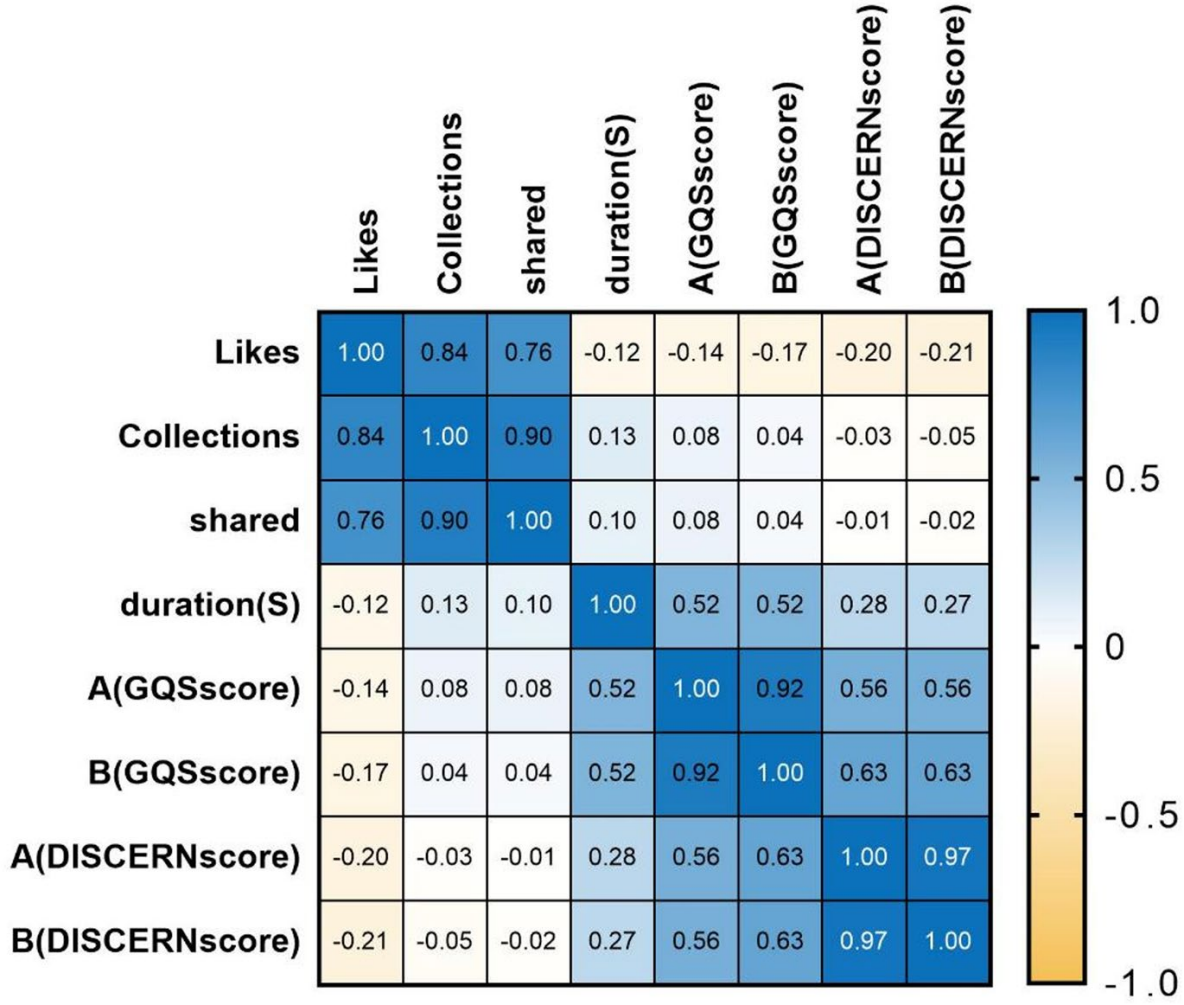
Spearman correlation analysis of video variables, GQS, and DISCERN scores for FNF videos.

A correlation heatmap was used to assess the relationships between engagement metrics (likes, collections, shares), video duration, and quality assessment scores (GQS/DISCERN) from two reviewers in Fig. 6. Firstly, the engagement metrics showed strong intercorrelations (*r* = 0.76–0.90). However, they had a minimal association with video duration (*r* = -0.12 to 0.13). Secondly, there was high inter-reviewer agreement, as evidenced by the strong correlations in scores. For GQS scores, the correlation was *r* = 0.92, and for DISCERN scores, it was *r* = 0.97. Moreover, both GQS and DISCERN scores demonstrated a moderate positive correlation with video duration. Specifically, for GQS scores, *r* = 0.52, and for DISCERN scores, *r* = 0.27–0.28. There were also significant associations between GQS and DISCERN scores (*r* = 0.56–0.63). Finally, the relationship between engagement metrics and quality scores was negligible (*r* = -0.21–0.08).

### Disease-specific clinical comprehensiveness and guideline adherence

**Fig. 7:**
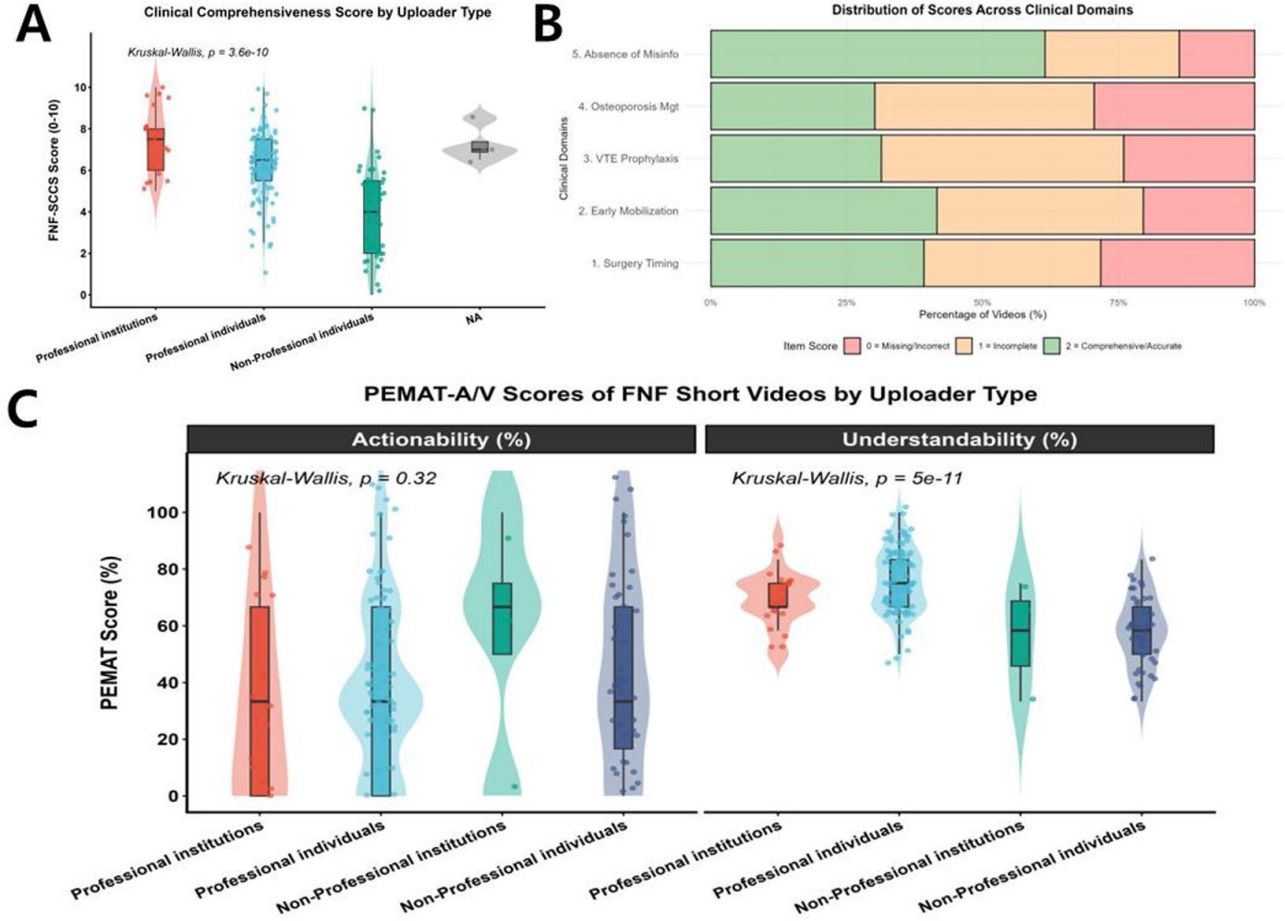
Disease-specific clinical comprehensiveness and PEMAT-A/V scores. (**A**) FNF-SCCS distribution by uploader type. (**B**) Proportion of videos meeting five core clinical domains. (**C**) PEMAT-A/V understandability and actionability scores by content theme.

The inter-rater reliability for the newly developed FNF-SCCS tool was excellent (ICC = 0.91). A Kruskal-Wallis test revealed significant differences in clinical depth and guideline adherence among different types of uploaders (*P* < 0.001). Specifically, videos published by professional institutions and professional individuals scored significantly higher on the FNF-SCCS compared to those from non-professional individuals (Fig. 7A).

Regarding the specific clinical domains, a concerning deficiency was observed in postoperative guidance. As illustrated in Figs. 7B and 24.1% of the evaluated videos completely failed to mention VTE prophylaxis (scored 0), and 29.5% neglected the importance of osteoporosis secondary prevention. Furthermore, explicit medical misinformation (scoring 0 on Item 5) was identified in 13.9% of the videos, which were predominantly uploaded by non-professional individuals.

### Understandability and actionability (PEMAT-A/V)

The evaluation using the PEMAT-A/V instrument also demonstrated high inter-rater agreement (ICC = 0.89). In terms of understandability, videos produced by professional individuals and institutions exhibited significantly higher scores (Median = 75.0%) compared to non-professionals (Median = 58.3%, *P* < 0.001), indicating clearer structure and better explanation of medical terminology.

Conversely, the overall actionability of the videos remained remarkably low across most uploader types, revealing a widespread lack of actionable, step-by-step instructions for patients. A notable exception was observed in videos categorized as “rehabilitation training,” which demonstrated significantly higher actionability scores (Median = 100.0%) compared to purely disease-knowledge or treatment-focused videos (Fig. 7C).

## Discussion

In recent years, the use of social media as a source of health-related information has grown remarkably, with individuals increasingly relying on these platforms for healthcare knowledge^22^. To better understand this trend in the context of FNF, we took several steps. Firstly, we systematically collected FNF-related videos from two major short-video platforms, TikTok and Bilibili. This approach overcomes the limitations of single-platform research and provides a more comprehensive view of the FNF information landscape on short-video platforms. Secondly, to ensure the reliability of our analysis, we employed multiple authoritative scoring tools to assess video quality. This enhanced the objectivity and accuracy of our research results. Finally, through our analysis, we aimed to identify the characteristics of FNF-related social media content in China. By doing so, we were able to pinpoint critical issues that have significant implications for patients, healthcare providers, and platform operators. Overall, these findings offer essential insights into the current state of FNF-related health information dissemination in the digital age.

### Short-video platforms as sources of femoral neck fracture (FNF) health information

In recent years, a substantial number of health-related videos have been disseminated on TikTok and Bilibili, one of China’s leading video-based education platforms, which have assumed an increasingly pivotal role in the dissemination of health information, prompting an inquiry into their content characteristics, quality, and origin^23,24^. This finding aligns with the growing role of social media in healthcare, where approximately 80% of internet users reportedly seek health-related information online^21,25^. With the global aging population, FNF incidence has significantly increased^26–28^. Given the profound impact of FNF on elderly individuals and their families, a large volume of related videos has emerged on social media platforms, offering convenient access to disease-related knowledge and partially satisfying public information needs. Moreover, our analysis found that the significant role of these platforms is underscored by the predominance of professional individuals as content creators, aligning with prior observations in orthopedic content analysis, suggesting healthcare professionals are increasingly leveraging social media for patient education^29^. However, with non-professional individuals contributing 25.9% of content, maintaining information quality control remains a persistent challenge on these platforms^30^. Notably, TikTok’s skewed distribution toward professional uploaders (71.6%) compared to Bilibili’s more balanced representation may reflect platform-specific user demographics and content moderation policies^31^. Despite similar content quality scores, the significantly higher engagement metrics (likes, collections, shares) observed on TikTok compared to Bilibili reflect findings from previous studies examining health information dissemination patterns^32,33^. This discrepancy suggests that platform algorithms and user behaviors might prioritize popularity over educational value, a phenomenon that is particularly concerning in orthopedic education, given the potential for misinformation to influence patient decision-making^34,35^.

Moreover, the absence of standardized oversight contributes to substantial inconsistencies in video quality and trustworthiness. Studies indicate that content produced by non-specialists frequently disseminates medically unsubstantiated claims or presents oversimplified explanations of complex conditions^36,37^. This phenomenon is particularly evident on short-video platforms. It has been noted in the literature that 40–60% of health-related content lacks proper scientific citations, potentially propagating misinformation^38^. For instance, an analysis of dermatological content demonstrated that 35% of eczema-related videos contained recommendations contradicting clinical guidelines^39^. Similarly, research on musculoskeletal conditions found that approximately 48% of osteoarthritis videos featured unverified treatment claims^12^. These findings underscore the critical need for quality control mechanisms in digital health communication.

### Information quality of FNF-short video

Our evaluation of FNF-related short videos on TikTok and Bilibili revealed significant quality deficiencies, with less than 20% of videos having GQS scores ≥ 4. This aligns with prior studies demonstrating that more than half of health-related short videos lack scientific rigor^40^. For example, a study on Turkish internet-based low back pain education materials—which found online patient education content in orthopedic-related areas often exhibits suboptimal quality, low reliability, and moderate readability that fails to meet public needs^10^. Notably, the consistency of these findings across different orthopedic diseases (FNF, low back pain or lumbar disc herniation etc.) underscores a common challenge in digital health communication for orthopedic topics, where scientific rigor and accessibility may be compromised^41^. Furthermore, the median duration of videos on both platforms is shorter, which fundamentally limits content depth, particularly for complex orthopedic topics requiring nuanced explanation^28,42^. Notably, disease knowledge videos scored highest: DISCERN = 4 and GQS = 4, respectively, while personal experience content performed the worst: DISCERN = 2 and GQS = 2, underscoring the risks of non-professional narratives^13,43^. Moreover, professional institutions consistently outperformed other uploaders. This quality gap stems from institutional advantages: multidisciplinary review processes, comprehensive coverage of treatment protocols, and adherence to clinical guidelines^16^. Paradoxically, non-professional institutions occasionally surpassed individual professionals in DISCERN scores, likely by repackaging professional content without adding value^30,36^. The 25.9% contribution from non-professional individuals remains concerning, as their videos showed higher engagement (median likes = 159.0) despite lower quality scores^32^. Furthermore, our disease-specific evaluation using the newly developed FNF-SCCS revealed critical gaps in clinical depth that generic tools like DISCERN might overlook. Notably, a significant proportion of videos failed to adhere to core clinical guidelines, particularly neglecting vital postoperative instructions such as VTE prophylaxis (24.1%) and osteoporosis secondary prevention (29.5%). Alarmingly, explicit medical misinformation was present in 13.9% of the content, predominantly originating from non-professionals. When assessing the “readability” and practical utility of these audiovisual materials using the PEMAT-A/V, we found that while professional videos possessed high understandability (median = 75.0%), effectively making complex medical jargon accessible to the public, the overall actionability was remarkably low. This indicates that most videos fail to provide patients with clear, step-by-step actionable instructions, with the notable exception of rehabilitation training videos.

To contextualize our platform focus, key differences exist between TikTok, Bilibili and other mainstream channels, such as the global video platform YouTube and AI-driven tools. Unlike AI tools (ChatGPT, Gemini, etc.), which generate text-based, query-specific responses often requiring high educational attainment for comprehension and exhibiting variable scientific rigor^44^, TikTok and Bilibili deliver visual-audio short-form content that is generally more accessible to FNF patients with diverse educational backgrounds. Regarding YouTube, a study on percutaneous tracheostomy-related content found that while professional videos maintain scientific rigor, non-professional content frequently contains misleading information^45^; this parallels our observations and is consistent with findings from another YouTube study on psoriasis, which reported that over half of non-professional videos disseminate misleading content^13^, highlighting that similar content quality issues exist across both popular local Chinese platforms and global English-dominated platforms. In comparison, online web channels typically offer structured, readable text with better quality control but lack visual engagement and suffer from lower user interaction^46^, whereas TikTok and Bilibili’s dynamic format drives higher engagement despite greater quality variability.

In short, these findings necessitate platform-level interventions. Algorithmic prioritization of content from verified institutions could leverage their demonstrated quality advantages^47^. Currently, medical associations should develop FNF-specific certification standards, particularly for rehabilitation training videos that show exceptional quality but limited availability^48,49^. Such measures could mitigate the current disconnect between engagement metrics and educational value^37^.

### Engagement metrics and video quality in FNF short videos

The correlation analysis yielded three significant findings. First, the strong inter-reviewer agreement (0.839 and 0.806) validates our assessment methodology and confirms the reliability of our quality metrics. The study found no significant correlation between FNF short video quality and engagement metrics, consistent with previous findings^50,51^. Commonly assumed to indicate better quality and appeal, high likes, collections, and shares do not guarantee such standards in FNF short videos or similar health-related content. TikTok users often struggle to distinguish high-quality videos from low-quality ones^52,53^. Additionally, the results may relate to TikTok and Bilibili users’ characteristics. As a lifestyle-entertainment app, its users favor entertaining content over highly credible material. Professional content, often less engaging and seen as dull, struggles to attract audiences or gain popularity on both platforms^54^. Therefore, experts should consider users’ needs and preferences when uploading videos, such as using simpler language and incorporating visual effects or animations. Platforms should also implement video filtering mechanisms to prioritize professional, high-quality content in search results, ensuring accurate knowledge dissemination.

### Correlation analysis

Spearman analysis showed strong correlations between likes, collections, and shares (all *r* > 0.76), suggesting popular videos get both collections and shares. Video duration moderately correlated with quality scores (*r* = 0.27–0.52). Notably, likes are weakly negatively correlated with quality metrics (*r* = -0.21 to -0.08), indicating popularity doesn’t guarantee quality. This aligns with the understanding that various factors on short-video platforms interact in complex ways, and user-engagement metrics do not always have a straightforward relationship with quality or other assessment-based scores.

## Limitations

### Study strengths

To provide a comprehensive overview of the research, the study’s key strengths include: Firstly, focus on an underexplored area of FNF patient education via short-video platforms. Secondly, use of validated assessment tools (The modified DISCERN tool was utilized to measure the information’s reliability, and the Global Quality Score (GQS) was employed to assess the videos’ overall quality.) with high inter-rater reliability (κ = 0.806–0.839). Beyond generic reliability tools (DISCERN and GQS), we pioneered the use of the disease-specific FNF-SCCS and the audiovisual-specific PEMAT-A/V, which provided unprecedented clinical depth regarding guideline adherence and understandability. Third, comprehensive coverage of China’s two most influential short-video platforms— TikTok and Bilibili— captures a representative sample of the Chinese digital health landscape, with data on uploader types, content themes, and user engagement offering multi-dimensional insights. Moreover, Practical relevance linking video quality to clinical outcomes, providing actionable recommendations for healthcare professionals, platform regulators, and patients.

### Study limitations

Firstly, the study exclusively analyzed content from TikTok and Bilibili. This selection was based on the study’s focus on the Chinese mainland context: these two platforms are the most widely used short-video platforms locally, with high penetration among all age populations and a large volume of FNF-related educational content. International platforms such as YouTube, Instagram, and Facebook were not included due to their limited market access and minimal user engagement in mainland China. As a result, the findings may not fully represent the diversity of health information across all global social media platforms. Second, all videos were in Chinese, and the platforms primarily serve Chinese users. This limits the generalizability of findings to other linguistic or cultural contexts where femoral neck fracture (FNF) education may differ in content or delivery. Third, the sample was limited to top-ranked videos per platform. While this simulates the real-world search behavior of patients—who rarely scroll past the top results—it may not represent the broader information ecosystem and is inherently susceptible to algorithmic bias. Additionally, the sample was limited to the top 100 videos per platform, potentially omitting less visible but clinically valuable content. Also, the cross-sectional design captures data at a single point in time. Social media content is dynamic, and trends in video quality or engagement may shift rapidly, necessitating longitudinal follow-up. Furthermore, regarding “readability”, we utilized the PEMAT-A/V to evaluate the understandability of audiovisual content; traditional text-based readability formulas (e.g., Flesch-Kincaid) were not applied as they are less suitable for Chinese short videos. Finally, while the DISCERN and GQS tools are validated, their application relies on reviewer judgment. Although inter-rater reliability was high (κ = 0.806–0.839), subtle biases in scoring subjective criteria may persist. In future studies, enhancing the scoring criteria could improve the accuracy and reliability of evaluations, and expanding the scope to include international platforms (where accessible) may provide a more global perspective on FNF-related health information dissemination.

## Conclusion

In this study, the reliability and quality of 164 FNF-related videos from two major short-video platforms (TikTok and Bilibili) were systematically evaluated. While these platforms provide accessible avenues for FNF education, the overall quality of content remains inconsistent. Regarding reliability (assessed via modified DISCERN), the median score was 3.00, with professional institutions achieving the highest reliability (median = 4.00) and non-professional individuals the lowest (median = 2.00); disease knowledge and rehabilitation training videos were more reliable (score = 4.00) than personal experience content (score = 2.00). Moreover, Bilibili showed superior reliability (40.9% of videos with DISCERN ≥ 4) compared to TikTok (23.2%). Crucially, disease-specific evaluations highlighted a concerning lack of clinical depth in the current digital ecosystem. A significant portion of videos omitted critical clinical guideline recommendations, such as VTE prophylaxis and osteoporosis management, and frequently lacked actionable instructions for patients, despite demonstrating acceptable understandability when produced by professionals. To address these challenges, collaborative efforts among platform regulators, healthcare professionals, and users are crucial for enhancing information quality and mitigating misinformation risks. Future studies should investigate longitudinal trends in video quality and conduct cross-cultural comparisons to optimize health communication strategies for FNF and other critical medical conditions.

## Supplementary Information

Below is the link to the electronic supplementary material.

Supplementary Material 1.

Supplementary Material 2.

Supplementary Material 3.

Supplementary Material 4.

## Data Availability

The raw data supporting the conclusions of this article will be made available by the corresponding author, without undue reservation, to any qualified researcher upon reasonable request. The data include details of the included short videos (e.g., title, uploader type, duration, engagement metrics) and the complete scoring results from the two reviewers using the modified DISCERN tool and Global Quality Score (GQS).

## Abbreviations

FNF: Femoral neck fractures
GQS: Global quality score

## References

[1] Dahl, O. E. & Pripp, A. H. Does the Risk of Death Within 48 Hours of Hip Hemiarthroplasty Differ Between Patients Treated with Cemented and Cementless Implants? A Meta-analysis of Large, National Registries. *Clin. Orthop. Relat. Res.* **480**, 343–350. 10.1097/corr.0000000000001952 (2022). 34491939 10.1097/CORR.0000000000001952 PMC8747483

[2] Cui, L., Zhao, S., Tian, H., Guo, W. & Dong, X. Curative efficacy of surgical procedures for older patients with femoral neck fracture: a network meta-analysis and systematic review. *J. Orthop. Surg. Res.* **17**, 127. 10.1186/s13018-022-02914-y (2022). 35236384 10.1186/s13018-022-02914-y PMC8889721

[3] Szymski, D. et al. Incidence and treatment of intracapsular femoral neck fractures in Germany. *Arch. Orthop. Trauma. Surg.* **143**, 2529–2537. 10.1007/s00402-022-04504-3 (2023). 35737120 10.1007/s00402-022-04504-3 PMC10110641

[4] Sekeitto, A. R., Sikhauli, N., van der Jagt, D. R., Mokete, L. & Pietrzak, J. R. T. The management of displaced femoral neck fractures: a narrative review. *EFORT Open. Rev.* **6**, 139–144. 10.1302/2058-5241.6.200036 (2021). 33828857 10.1302/2058-5241.6.200036 PMC8022011

[5] Huang, M. N. et al. The content quality and educational significance of early childhood caries on short video platforms. *BMC Public. Health*. **25**, 1713. 10.1186/s12889-025-22962-3 (2025). 40346611 10.1186/s12889-025-22962-3 PMC12063300

[6] Guan, J. L. et al. Videos in Short-Video Sharing Platforms as Sources of Information on Colorectal Polyps: Cross-Sectional Content Analysis Study. *J. Med. Internet Res.* **26**, e51655. 10.2196/51655 (2024). 39470708 10.2196/51655 PMC11558218

[7] Ozduran, E. & Hanci, V. Youtube as a source of information about stroke rehabilitation during the COVID-19 pandemic. *Neurology Asia* **28** (2023).

[8] Özbek, İ. C., Hancı, V. & Özduran, E. Digital Guidance: Quality and Readability Analysis of Artificial Intelligence-Generated Spondyloarthropathy Texts. *Turkish J. Osteoporosis* (2025).

[9] Iikuni, N. et al. Safety and effectiveness profile of raloxifene in long-term, prospective, postmarketing surveillance. *J. Bone Min. Metab.* **30**, 674–682. 10.1007/s00774-012-0365-1 (2012). 10.1007/s00774-012-0365-1 22752125

[10] Ozduran, E., Hanci, V. & Erkin, Y. Evaluating the readability, quality and reliability of online patient education materials on chronic low back pain. *Natl. Med. J. India*. **37**, 124–130. 10.25259/nmji_327_2022 (2024). 39399994 10.25259/NMJI_327_2022

[11] He, F. et al. Quality and reliability of pediatric pneumonia related short videos on mainstream platforms: cross-sectional study. *BMC Public. Health*. **25**, 1896. 10.1186/s12889-025-22963-2 (2025). 40410758 10.1186/s12889-025-22963-2 PMC12101000

[12] Hong, T. I. et al. Analysis of the Perception and Treatment of Osteoarthritis of the Knee Through Social Media: An Observational Study of the Top 100 Viral TikTok Videos. *Cureus* 15, e48487 (2023). 10.7759/cureus.48487 10.7759/cureus.48487 PMC10630902 38024061

[13] Mueller, S. M. et al. The Absence of Evidence is Evidence of Non-Sense: Cross-Sectional Study on the Quality of Psoriasis-Related Videos on YouTube and Their Reception by Health Seekers. *J. Med. Internet Res.* **21**, e11935. 10.2196/11935 (2019). 30664460 10.2196/11935 PMC6357908

[14] Kaplan, K. & Solak, Y. Evaluation of YouTube Videos on Hepatocellular Carcinoma. *J. Korean Med. Sci.* **38**, e50. 10.3346/jkms.2023.38.e50 (2023). 36808545 10.3346/jkms.2023.38.e50 PMC9941019

[15] Venosa, M. et al. Stem Cells in Orthopedic Web Information: An Assessment with the DISCERN Tool. *Cartilage* 13, 519s-525s (2021). 10.1177/19476035211040161 10.1177/19476035211040161 PMC8808862 34425692

[16] Charnock, D., Shepperd, S., Needham, G. & Gann, R. DISCERN: an instrument for judging the quality of written consumer health information on treatment choices. *J. Epidemiol. Community Health*. **53**, 105–111. 10.1136/jech.53.2.105 (1999). 10396471 10.1136/jech.53.2.105 PMC1756830

[17] Bernard, A. et al. A systematic review of patient inflammatory bowel disease information resources on the World Wide Web. *Am. J. Gastroenterol.* **102**, 2070–2077. 10.1111/j.1572-0241.2007.01325.x (2007). 17511753 10.1111/j.1572-0241.2007.01325.x

[18] Stambough, J. B. et al. Clinical Practice Guidelines in Action: Differences in Femoral Neck Fracture Management by Trauma and Arthroplasty Training. *J. Am. Acad. Orthop. Surg.* **27**, 287–294. 10.5435/jaaos-d-17-00760 (2019). 30278016 10.5435/JAAOS-D-17-00760

[19] Fishlock, A., Scarsbrook, C. & Marsh, R. Adherence to guidelines regarding total hip replacement for fractured neck of femur. *Ann. R Coll. Surg. Engl.* **98**, 422–424. 10.1308/rcsann.2016.0151 (2016). 27092581 10.1308/rcsann.2016.0151 PMC5209976

[20] Cui, N. et al. Quality Assessment of TikTok as a Source of Information About Mitral Valve Regurgitation in China: Cross-Sectional Study. *J. Med. Internet Res.* **26**, e55403. 10.2196/55403 (2024). 39163110 10.2196/55403 PMC11372326

[21] Grajales, F. J. 3, Sheps, S., Ho, K., Novak-Lauscher, H., Eysenbach, G. & rd,, & Social media: a review and tutorial of applications in medicine and health care. *J. Med. Internet Res.* **16**, e13. 10.2196/jmir.2912 (2014). 24518354 10.2196/jmir.2912 PMC3936280

[22] Xiao, L. et al. Public’s preferences for health science popularization short videos in China: a discrete choice experiment. *Front. Public. Health*. **11**, 1160629. 10.3389/fpubh.2023.1160629 (2023). 37601206 10.3389/fpubh.2023.1160629 PMC10436607

[23] Chen, Z., Pan, S. & Zuo, S. TikTok and YouTube as sources of information on anal fissure: A comparative analysis. *Front. Public. Health*. **10**, 1000338. 10.3389/fpubh.2022.1000338 (2022). 36407987 10.3389/fpubh.2022.1000338 PMC9669434

[24] Muenster, R. M., Gangi, K. & Margolin, D. Alternative Health and Conventional Medicine Discourse About Cancer on TikTok: Computer Vision Analysis of TikTok Videos. *J. Med. Internet Res.* **26**, e60283. 10.2196/60283 (2024). 39652864 10.2196/60283 PMC11667741

[25] Signorini, A., Segre, A. M. & Polgreen, P. M. The use of Twitter to track levels of disease activity and public concern in the U.S. during the influenza A H1N1 pandemic. *PLoS One*. **6**, e19467. 10.1371/journal.pone.0019467 (2011). 21573238 10.1371/journal.pone.0019467 PMC3087759

[26] Bogoch, E. et al. High Rates of Imminent Subsequent Fracture After Femoral Neck Fracture in the Elderly. *J. Bone Joint Surg. Am.* **104**, 1984–1992. 10.2106/jbjs.22.00088 (2022). 36017942 10.2106/JBJS.22.00088

[27] Morrissey, N., Iliopoulos, E., Osmani, A. W. & Newman, K. Neck of femur fractures in the elderly: Does every hour to surgery count? *Injury* **48**, 1155–1158. (2017). 10.1016/j.injury.2017.03.007 28325670 10.1016/j.injury.2017.03.007

[28] Bigoni, M. et al. Internal fixation of intracapsular femoral neck fractures in elderly patients: mortality and reoperation rate. *Aging Clin. Exp. Res.* **32**, 1173–1178. 10.1007/s40520-019-01237-z (2020). 31175608 10.1007/s40520-019-01237-z

[29] Youssef, Y. et al. Social media and internet use among orthopedic patients in Germany-a multicenter survey. *Front. Digit. Health*. **7**, 1486296. 10.3389/fdgth.2025.1486296 (2025). 40297730 10.3389/fdgth.2025.1486296 PMC12035442

[30] Zhang, R. et al. Analyzing dissemination, quality, and reliability of Chinese brain tumor-related short videos on TikTok and Bilibili: a cross-sectional study. *Front. Neurol.* **15**, 1404038. 10.3389/fneur.2024.1404038 (2024). 39494168 10.3389/fneur.2024.1404038 PMC11527622

[31] Zhao, X., Yao, X., Sui, B. & Zhou, Y. Current status of short video as a source of information on lung cancer: a cross-sectional content analysis study. *Front. Oncol.* **14**, 1420976. 10.3389/fonc.2024.1420976 (2024). 39650058 10.3389/fonc.2024.1420976 PMC11621006

[32] Zheng, S. et al. Quality and Reliability of Liver Cancer-Related Short Chinese Videos on TikTok and Bilibili: Cross-Sectional Content Analysis Study. *J. Med. Internet Res.* **25**, e47210. 10.2196/47210 (2023). 37405825 10.2196/47210 PMC10357314

[33] Song, S. et al. Short-Video Apps as a Health Information Source for Chronic Obstructive Pulmonary Disease: Information Quality Assessment of TikTok Videos. *J. Med. Internet Res.* **23**, e28318. 10.2196/28318 (2021). 34931996 10.2196/28318 PMC8726035

[34] Swire-Thompson, B. & Lazer, D. Public Health and Online Misinformation: Challenges and Recommendations. *Annu. Rev. Public. Health*. **41**, 433–451. 10.1146/annurev-publhealth-040119-094127 (2020). 31874069 10.1146/annurev-publhealth-040119-094127

[35] Walsh-Buhi, E. R. Social Media and Cancer Misinformation: Additional Platforms to Explore. *Am. J. Public. Health*. **110**, S292–s293. 10.2105/ajph.2020.305949 (2020). 33001721 10.2105/AJPH.2020.305949 PMC7532333

[36] Kouvari, M. et al. Digital Health Interventions for Weight Management in Children and Adolescents: Systematic Review and Meta-analysis. *J. Med. Internet Res.* **24**, e30675. 10.2196/30675 (2022). 35156934 10.2196/30675 PMC8887634

[37] Hause, A. M. et al. Safety Monitoring of Bivalent COVID-19 mRNA Vaccine Booster Doses Among Children Aged 5–11 Years - United States, October 12-January 1, 2023. *MMWR Morb Mortal. Wkly. Rep.* **72**, 39–43. 10.15585/mmwr.mm7202a5 (2023). 36634021 10.15585/mmwr.mm7202a5 PMC9869731

[38] Tschandl, P. Risk of Bias and Error From Data Sets Used for Dermatologic Artificial Intelligence. *JAMA Dermatol.* **157**, 1271–1273. 10.1001/jamadermatol.2021.3128 (2021). 34550304 10.1001/jamadermatol.2021.3128

[39] Mueller, S. M. et al. Fiction, Falsehoods, and Few Facts: Cross-Sectional Study on the Content-Related Quality of Atopic Eczema-Related Videos on YouTube. *J. Med. Internet Res.* **22**, e15599. 10.2196/15599 (2020). 32329744 10.2196/15599 PMC7210495

[40] Amezcua, L., Rivera, V. M., Vazquez, T. C., Baezconde-Garbanati, L. & Langer-Gould, A. Health Disparities, Inequities, and Social Determinants of Health in Multiple Sclerosis and Related Disorders in the US: A Review. *JAMA Neurol.* **78**, 1515–1524. 10.1001/jamaneurol.2021.3416 (2021). 34605866 10.1001/jamaneurol.2021.3416

[41] Wang, Y., Lian, S., Liao, L. & Wang, W. Assessment of information quality and reliability of short videos related to lumbar disc herniation on selected video platforms. *Digit. Health*. **11**, 20552076251393278. 10.1177/20552076251393278 (2025). 41181560 10.1177/20552076251393278 PMC12576092

[42] Shi, P., Li, N., Zhou, S. & Hua, K. Letter to the Editor: Does the Risk of Death Within 48 Hours of Hip Hemiarthroplasty Differ Between Patients Treated With Cemented and Cementless Implants? A Meta-analysis of Large, National Registries. *Clin. Orthop. Relat. Res.* **480**, 2468–2469. 10.1097/corr.0000000000002443 (2022). 36374582 10.1097/CORR.0000000000002443 PMC10538899

[43] Joshi, M. et al. Assessment of quality and reliability of YouTube videos for patient and physician education on inflammatory myositis. *Clin. Rheumatol.* **42**, 1339–1349. 10.1007/s10067-023-06522-x (2023). 36759401 10.1007/s10067-023-06522-x PMC9910767

[44] Ozduran, E., Akkoc, I., Büyükçoban, S., Erkin, Y. & Hanci, V. Readability, reliability and quality of responses generated by ChatGPT, gemini, and perplexity for the most frequently asked questions about pain. *Med. (Baltim).* **104**, e41780. 10.1097/md.0000000000041780 (2025). 10.1097/MD.0000000000041780 PMC11922396 40101096

[45] Hancı, V., Öner, Ö., Özduran, E. & Gökel, E. Youtube as a source of information about Percutan Tracheostomy. *Gazi Med. J.* **34**, 4 (2023).

[46] Özduran, E. & Hanci, V. Evaluating the readability, quality and reliability of online information on Behçet’s disease. *Reumatismo*10.4081/reumatismo.2022.1495 (2022). 36101989 10.4081/reumatismo.2022.1495

[47] Moorhead, S. A. et al. A new dimension of health care: systematic review of the uses, benefits, and limitations of social media for health communication. *J. Med. Internet Res.* **15**, e85. 10.2196/jmir.1933 (2013). 23615206 10.2196/jmir.1933 PMC3636326

[48] Pereira, K., Phillips, B., Johnson, C. & Vorderstrasse, A. Internet delivered diabetes self-management education: a review. *Diabetes Technol. Ther.* **17**, 55–63. 10.1089/dia.2014.0155 (2015). 25238257 10.1089/dia.2014.0155

[49] Burkow, T. M. et al. Internet-enabled pulmonary rehabilitation and diabetes education in group settings at home: a preliminary study of patient acceptability. *BMC Med. Inf. Decis. Mak.* **13**, 33. 10.1186/1472-6947-13-33 (2013). 10.1186/1472-6947-13-33 PMC3599897 23496829

[50] Yang, S., Zhan, J. & Xu, X. Is TikTok a high-quality source of information on thyroid cancer? *Endocrine* **81**, 270–276. 10.1007/s12020-023-03332-8 (2023). 36840912 10.1007/s12020-023-03332-8

[51] Kim, M. J., Kim, J. R., Jo, J. H., Kim, J. S. & Park, J. W. Temporomandibular disorders-related videos on YouTube are unreliable sources of medical information: A cross-sectional analysis of quality and content. *Digit. Health*. **9**, 20552076231154377. 10.1177/20552076231154377 (2023). 36762021 10.1177/20552076231154377 PMC9903026

[52] Sun, F., Zheng, S. & Wu, J. Quality of Information in Gallstone Disease Videos on TikTok: Cross-sectional Study. *J. Med. Internet Res.* **25**, e39162. 10.2196/39162 (2023). 36753307 10.2196/39162 PMC9947761

[53] Li, W. S. et al. Investigation and quality evaluation of Internet videos related to diet therapy for chronic pancreatitis. *Med. (Baltim).* **104**, e42523. 10.1097/md.0000000000042523 (2025). 10.1097/MD.0000000000042523 PMC12091591 40388769

[54] Ding, R. et al. Health information in short videos about metabolic dysfunction-associated steatotic liver disease: Analysing quality and reliability. *Liver Int.* **44**, 1373–1382. 10.1111/liv.15871 (2024). 38441405 10.1111/liv.15871

