# Supplementary material for: Quality and reliability of femoral neck fracture educational short videos: a cross-sectional study
Source: Sci Rep. 2026 Mar 30;16:10652. doi: 10.1038/s41598-026-46431-y (PMC13040079; doi:10.1038/s41598-026-46431-y)
Supplement: Supplementary file 1 — Supplementary Material 1. [file 41598_2026_46431_MOESM1_ESM.docx]

**Supplementary: Global Quality Score (GQS) (Scoring ranges from 1 to 5)**

| **GQS Definition** | **Score** |
| --- | --- |
| Poor quality：Specifically, the content is illogical, the mobility is poor, most of the information is missing, and it is useless for patients. | 1 |
| Generally poor quality ：the content logic is poor, although some information is listed, more important information is still missing, and the use of patients is very limited. | 2 |
| Moderate quality：some important information is adequately discussed. | 3 |
| Good quality and flow：Specifically, the video logic is clear and smooth, covering most of the relevant information, which is useful for patients. | 4 |
| Excellent quality and flow：Specifically, the video logic is clear, and the content is very smooth, which is very useful for patients. | 5 |
