# Supplementary material for: Quality and reliability of femoral neck fracture educational short videos: a cross-sectional study
Source: Sci Rep. 2026 Mar 30;16:10652. doi: 10.1038/s41598-026-46431-y (PMC13040079; doi:10.1038/s41598-026-46431-y)
Supplement: Supplementary file 2 — Supplementary Material 2. [file 41598_2026_46431_MOESM2_ESM.docx]

**Supplementary: Modified DISCERN quality criteria for assessing the reliability of video. (1 point for answer ‘yes’, 0 point for answer ‘no’)**

| **Reliability Score** |
| --- |
| 1. Is the video clear, concise, and understandable? |
| 2. Are valid sources cited? |
| 3. Is the content presented balanced and unbiased? |
| 4. Are additional sources of content listed for patient reference? |
| 5. Are areas of uncertainty mentioned? |
