# Supplementary material for: Quality and reliability of femoral neck fracture educational short videos: a cross-sectional study
Source: Sci Rep. 2026 Mar 30;16:10652. doi: 10.1038/s41598-026-46431-y (PMC13040079; doi:10.1038/s41598-026-46431-y)
Supplement: Supplementary file 3 — Supplementary Material 3. [file 41598_2026_46431_MOESM3_ESM.docx]

**Supplementary File 3. Femoral Neck Fracture-Specific Clinical Comprehensiveness Score (FNF-SCCS)**

***Scoring domains, clinical rationale, and rating criteria for the FNF-SCCS instrument.***

| **Assessment Domain** | **Core Clinical Relevance** | **Scoring Criteria** |
| --- | --- | --- |
| **1. Timing of Surgery** | Clinical practice guidelines for femoral neck fracture, particularly in older adults, strongly recommend early surgical intervention (e.g., within 48 hours) to reduce mortality and perioperative complications. | 0: Surgery is not mentioned, or incorrect advice is provided (e.g., recommending conservative treatment or prolonged bed rest for older patients without contraindications to surgery). 1: Surgery is mentioned, but the urgency of timely intervention is not emphasized and no specific time window is provided. 2: The content clearly recommends early surgery (e.g., “as soon as possible” or “within 48 hours”) and/or explains the risks associated with delayed surgery. |
| **2. Early Mobilization and Rehabilitation** | Early postoperative mobilization and weight-bearing are essential for functional recovery, whereas prolonged immobilization can be life-threatening in older patients. | 0: Postoperative rehabilitation is not mentioned, or clearly incorrect advice is given (e.g., complete bed rest for an extended period). 1: Rehabilitation or physical therapy is mentioned, but the recommendation is vague and lacks actionable guidance. 2: The content explicitly encourages early ambulation or early weight-bearing after surgery, and/or demonstrates appropriate bedside rehabilitation exercises (e.g., straight-leg raise or hip-flexion exercises). |
| **3. Venous Thromboembolism (VTE) Prophylaxis** | Major orthopedic surgery carries a high risk of deep vein thrombosis and pulmonary embolism, which are among the leading causes of perioperative morbidity and mortality. | 0: Thromboembolic risk or prevention is not mentioned at all. 1: The risk of thrombosis is acknowledged, but no specific preventive strategy is provided. 2: Specific preventive measures are accurately described, such as ankle-pump exercises, compression stockings, or anticoagulant therapy (e.g., heparin). |
| **4. Osteoporosis Management and Secondary Prevention** | In older adults, femoral neck fractures are commonly fragility fractures; without systematic osteoporosis management, the risk of subsequent contralateral fracture remains high. | 0: Osteoporosis or calcium/vitamin supplementation is not mentioned. 1: Osteoporosis is mentioned as a cause, but no long-term management or secondary prevention strategy is provided. 2: The content explicitly recommends structured anti-osteoporosis treatment after surgery, including standard calcium and vitamin D supplementation and/or prescription anti-osteoporosis medications. |
| **5. Absence of Misinformation or Pseudoscientific Claims** | Short-video platforms frequently contain folk remedies and unsupported claims; screening for misinformation is therefore a minimum requirement for evaluating content quality. | 0: The video contains seriously misleading information that clearly contradicts modern medical knowledge (e.g., “bone broth can heal the fracture,” “older adults must never undergo surgery,” or “a folk remedy can cure all cases”). 1: The information contains minor flaws, exaggeration, outdated claims, or a strong commercial marketing bias, but is unlikely to cause immediate harm. 2: No misleading or pseudoscientific information is present, and the content is fully consistent with contemporary evidence-based medical consensus. |

**Notes:**

**Abbreviations:** FNF-SCCS, Femoral Neck Fracture-Specific Clinical Comprehensiveness Score; AAOS, American Academy of Orthopaedic Surgeons; NICE, National Institute for Health and Care Excellence; VTE, Venous Thromboembolism.

**Clinical Rationale:** The selection of these five domains is based on the consensus of major orthopedic clinical practice guidelines. These metrics specifically measure the degree to which short-form video content aligns with evidence-based surgical and postoperative management protocols for elderly hip fractures.

**Scoring Logic:** Each video was independently evaluated by two senior orthopedic surgeons. A score of 0 indicates the complete omission or clinical inaccuracy of the information; 1 indicates the information is mentioned but lacks specific detail; 2 indicates the content is both accurate and sufficiently detailed to guide patient self-management.

**Reliability:** Inter-rater reliability for this scale was assessed using the Intraclass Correlation Coefficient (ICC).
