# Supplementary material for: Quality and reliability of femoral neck fracture educational short videos: a cross-sectional study
Source: Sci Rep. 2026 Mar 30;16:10652. doi: 10.1038/s41598-026-46431-y (PMC13040079; doi:10.1038/s41598-026-46431-y)
Supplement: Supplementary file 4 — Supplementary Material 4. [file 41598_2026_46431_MOESM4_ESM.docx]

**Supplementary File 4.** **The scoring criteria for Patient Education Materials Assessment Tool for Audiovisual Materials (PEMAT-A/V).**

| **Items (Total =17)** | **Options** |
| --- | --- |
| **Understandability** |  |
| Question 1. The material makes its purpose completely evident. | 0, 1 |
| Question 3. The material uses common, everyday language. | 0, 1 |
| Question 4. Medical terms are used only to familiarize audience with the terms. When used, medical terms are defined. | 0, 1 |
| Question 5. The material uses the active voice. | 0, 1 |
| Question 8. The material breaks or “chunks” information into short sections. | 0, 1, NA |
| Question 9. The material’s sections have informative headers. | 0, 1, NA |
| Question 10. The material presents information in a logical sequence. | 0, 1 |
| Question 11. The material provides a summary. | 0, 1, NA |
| Question 12. The material uses visual cues (e.g., arrows, boxes, bullets, bold, larger font, highlighting) to draw attention to key points. | 0, 1, NA |
| Question 13. Text on screen is easy to read. | 0, 1, NA |
| Question 14. The material allows the user to hear the words clearly (e.g., not too fast, not garbled). | 0, 1, NA |
| Question 18. The material uses illustrations and photographs that are clear and uncluttered. | 0, 1, NA |
| Question 19. The material uses simple tables with short and clear row and column headings. | 0, 1, NA |
| **Actionability** |  |
| Question 20. The material clearly identifies at least one action the user can take. | 0, 1 |
| Question 21. The material addresses the user directly when describing actions. | 0, 1 |
| Question 22. The material breaks down any action into manageable, explicit steps. | 0, 1 |
| Question 25. The material explains how to use the charts, graphs, tables or diagrams to take actions. | 0, 1, NA |

*Disagree=0 Agree=1 NA=Not Applicable. Since PEMAT is numbered according to the items for printable materials, questions 2, 6, 7, 15, 16, 17, 23, and 24 are missing in PEMAT-A/V.
